# Has stopping stopped making sense? Disentangling the processes elicited by complex action cancellation paradigms

**DOI:** 10.3758/s13415-026-01440-2

**Published:** 2026-04-09

**Authors:** Simon Weber, Sarah A. Kemp, Mark R. Hinder

**Affiliations:** 1 Sensorimotor Neuroscience and Aging Research Laboratory, University of Tasmania, Hobart, TAS 7005 Australia; 2 Amsterdam Mathematical Psychology Lab, University of Amsterdam, Amsterdam, Netherlands

**Keywords:** Inhibition, Action cancellation, Response inhibition, Stop signal task, Selective stopping, Pause then cancel, Stop signal reaction time

## Abstract

Action cancellation represents a fundamental aspect of human motor control. Nonetheless, the behavioural paradigms used to study it are methodologically diverse and capture an incompletely understood combination of processes. Advancing theoretical accounts of action cancellation have motivated increasingly complicated experimental designs, leading some authors to draw comparisons between “simple” and “complex” stopping tasks. The latter describes a mix of paradigms, which variably require increased working memory demands, stimulus discrimination, distractor interference, or more complex stimulus–response mappings. This narrative review provides an accessible explanation of commonly implemented complex stopping tasks, selected based on their relevance to current theoretical debates. Throughout, we describe the challenges associated with interpreting performance in these tasks and highlight future directions for research. Notably, much of the past work using these tasks has applied traditional methods of indexing the speed of stopping, which were developed specifically for *simple* stopping tasks, and do not generalise well beyond this context. Recent research using cognitive modelling and physiological approaches has failed to replicate earlier findings, raising fundamental questions regarding how action cancellation should be indexed. Furthermore, physiological observations in complex stopping tasks appear to be incompatible with current accounts of the neurological mechanisms that underpin action cancellation. We propose potential ways forward, highlighting how the current lack of well-controlled comparisons between task types means the literature risks extensive task-specific findings that fail to generalise between laboratories, and—critically—beyond the laboratory.

Action cancellation describes the process of halting or modifying prepotent or initiated actions in response to changing environmental demands. This fundamental motor capacity is known to slow as we age (Rey-Mermet & Gade, 2018; Williams et al., 1999) and to be impaired in a range of clinical conditions (Lipszyc & Schachar, 2010). However, the behavioural paradigms used to assess this process are methodologically diverse and capture an incompletely understood combination of processes. Reactively cancelling a movement in response to an unexpected stimulus requires attentional orienting, stimulus appraisal (to determine if stopping is required), response selection, and potentially a combination of both voluntary and involuntary suppression of motor output. Recent attempts to meaningfully dissociate these subprocesses, and to assess their generalisability to other contexts, has motivated the development of increasingly complex behavioural paradigms. The heterogeneous array of task designs in the literature has led some authors to draw comparisons between “simple” and “complex” stopping tasks (Aziz-Safaie, 2024), with the latter describing a mix of tasks which variably require increased working memory demands, perceptual decision making and/or more complex stimulus–response mappings, relative to more simple variants. By presenting an accessible overview of commonly implemented complex stopping tasks, we aim to provide a coherent reference point for their interpretation and the theoretical questions motivating them. Beginning with simple stopping tasks, we highlight how, even amongst these simple variants, fundamental uncertainties exist regarding a) the underlying processes being assessed, and b) how task design impacts performance.

## Eliciting and interpreting action cancellation

### Action cancellation as a component of inhibition

Research investigating action cancellation typically contextualises it as part of the broader construct of “inhibition” (also referred to as “inhibitory control”), which constitutes a well-established field of study in cognitive neuroscience (Bari & Robbins, 2013). While inhibition is multifaceted and numerous differing taxonomies have been proposed (Friedman & Miyake, 2004; Nigg, 2000; Tiego et al., 2018), most contemporary research draws a distinction between a) the ability to ignore distractor information (variably referred to as “interference control,” “interference suppression,” “perceptual inhibition,” or “attentional inhibition”), and b) the ability to inhibit dominant motor responses (further divided into “action cancellation” and “action restraint”; described below). This distinction is supported by factor analyses (Rey-Mermet et al., 2018; Shields & Yonelinas, 2025; Tiego et al., 2018) and dissociable neural underpinnings (Sebastian et al., 2013). The current review regards the mechanisms underpinning action cancellation, which have traditionally been investigated with two major behavioural tasks: the go no-go task (GNGT) and the stop signal task (SST).

The GNGT and SST both have an extensive history in psychological research (Donders, 1969; Lappin & Eriksen, 1966; Vince, 1948). Trials in a GNGT require a participant to *either* enact a movement (“go trial”) or refrain from making a movement (“no-go trial”), based on the stimulus that appears (either a “go” stimulus, or a “no-go” / “stop” stimulus). In contrast, the SST features a go stimulus on *every* trial, with a subset of trials *additionally* presenting a stop stimulus after the go stimulus, following a variable delay (stop signal delay [SSD]). As such, in the GNGT, the participant is rapidly choosing between two options following stimulus onset (to respond or not respond) while in the SST the participant is instructed to initiate a response in every trial (to the go signal) and attempt to cancel this response should the infrequent stop signal occur.

Factor analyses examining performance on a broad range of inhibitory tasks suggest that performance in GNGTs and SSTs strongly load onto a single factor (Bender et al., 2016; Shields & Yonelinas, 2025; Tiego et al., 2018). However, behavioural evidence suggests a dissociation between the two (Littman & Takács, 2017), and brain imaging suggests they have overlapping, but distinct, neural substrates (Sebastian et al., 2013; Swick et al., 2011; Zhang et al., 2017). As such, the GNGT is typically seen as an index of action *restraint* while the SST is seen as a measure of action *cancellation* (Schachar et al., 2007).^1^ Further supporting this distinction, electrophysiological research observes distinct signatures and temporal dynamics in these two tasks (Raud et al., 2020). It has been proposed that action *cancellation* may only occur during GNGTs when no-go trials are in the minority, and trials occur at a rapid rate (Wessel, 2018). In sum, while GNGTs offer a means of assessing aspects of inhibitory control (in particular, action restraint), we focus specifically on action cancellation, which is more consistently and reliably elicited by SSTs.

### Indexing action cancellation in stop signal tasks

Behavioural outcomes in the SST have historically been interpreted using a conceptual “race” between two distinct processes: the go process, triggered by the go signal, and the stop process, triggered by the stop signal (Logan et al., 1984). Whichever of these two processes crosses the response threshold first determines the winner of the race and dictates whether or not a response is made. The primary benefit of this conception is that it allows for the estimation of the finishing time of the stop process, termed stop signal reaction time (SSRT),^2^ which is not directly observable in behavioural data. While SSRT represents an indirect estimate of an abstracted process, rather than the speed of a specific biological mechanism, many studies have sought associations between this value and specific brain regions/processes. For instance, functional imaging studies have demonstrated correlations between SSRT and activity in the presupplementary motor area and inferior frontal gyrus (Cai et al., 2014; Rubia et al., 2007). Brain stimulation studies perturbing these regions have also demonstrated specific slowing of SSRT, with no effect on the speed of action initiation (Sundby et al., 2021; Chen et al., 2009). Stop signal reaction time has also been used as a benchmark when assessing the validity of electrophysiological signatures thought to be associated with the stop process (Hervault et al., 2025; Huster et al., 2020). Beyond these associations, the fact that SSRT can be used to quantify slowed action cancellation as a function of healthy ageing (Rey-Mermet & Gade, 2018) and in many clinical conditions (Lipszyc & Schachar, 2010) has no doubt facilitated its establishment as the gold standard measure of stopping speed. Nonetheless, it remains that SSRT is an estimate derived from behavioural data and is in itself not reflective of a specific brain mechanism.

While SSRT provides an estimate of the speed of a stopping process, a number of assumptions must be met for its application to be valid (Verbruggen et al., 2019). For example, an assumption of the race model is that, for any given participant, average response times (RTs) on failed stop trials is faster than average RTs for go trials (because, if the model is correct, failed stops should represent trials with faster go processes—those that were more difficult to inhibit before a response, e.g., a button press, occurred). Cases where this assumption is violated suggest an interaction between go and stop processes (e.g., that the stop signal had a *slowing* effect on the go process, rather than just operating in parallel and beating it in an independent “race”). Violations of this assumption can be observed over a range of SST tasks, especially at short SSDs (Bissett et al., 2021a, 2021b; Gulberti et al., 2014). Other shortcomings of traditional SSRT estimates include their failure to account for attentional influences: instances where the go process is not initiated (resulting in a successful “stop” by virtue of the go process not being in the race), or where the stop process fails to be triggered by the stop signal (trigger failures), resulting in the go process winning without competition (Skippen et al., 2020). Although infrequent, these trials greatly bias SSRT estimates (for more details, see Doekemeijer et al., 2021; Matzke et al., 2017).

Numerous solutions have been proposed to these problems, for instance, by removing trials or participants that demonstrate clear violations of the above assumptions (Bissett et al., 2021a, 2021b), or via modelling approaches. Specifically, the BEESTS (Bayesian ex-Gaussian estimation of stop-signal RT distributions) modelling approach estimates a distribution of SSRTs, rather than a single measure (Matzke et al., 2013, 2017). The distribution (assumed to be ex-Gaussian) is described with three parameters: the mean (μ) and standard deviation (σ) of the Gaussian component, and τ describing the ex-Gaussian component (the rightward tail). Thus, greater μ indicates *slower* stopping speeds, while greater σ or τ suggest greater *variability* in the speed of the stop process. Decomposing SSRT estimates into these distinct parameters has allowed for insights into which subprocesses underpin SST performance differences in clinical populations. For example, while children with ADHD do not demonstrate slower μ than healthy controls (that is, the *speed* of the stop process is not different; Weigard et al., 2019), τ is greater, suggesting that differences are driven by attentional lapses, rather than the action cancellation process itself. In sum, while recent advances in cognitive modelling have enriched interpretations of behavioural data from SSTs, they also highlight that performance on SSTs is not governed by a unitary process and that the differences in SSRT that have historically been attributed to meaningful difference in action cancellation, may instead result from changes to other processes, e.g., sustained attention. Importantly, in the context of the current review, such computational models have not yet been developed for many of the more complex variants of the SST, which require a far greater number and variety of processes.

An alternative to estimating the speed of latent stop processes based on the presence or absence of behavioural responses is to directly record task-relevant muscle activity using electromyography (EMG; De Jong et al., 1990; Raud & Huster, 2017; Salomoni et al., 2025). A subset of successful stop trials will typically show some form of muscle activity in the muscles responsible for issuing the response (e.g., finger flexors/abductors during button presses), which is suppressed before an overt behavioural response is generated. The delay between the appearance of the stop signal and the time at which muscle activity of an initiated response begins to decrease can be directly measured, allowing a *single-trial* measure of stopping speed (Jana et al., 2020; Raud et al., 2022).^3^ This approach has been extended to assess both agonist and antagonist muscles (Atsma et al., 2018) and for a range of upper limb movements (De Havas, et al., 2020). Deriving single trial measures of stopping speed at the muscle circumvents many of the limitations associated with SSRT calculation, and removes the ballistic stage of action cancellation (i.e., mechanical delays between muscle contraction and the ensuing button press being registered), which are subject to variations in experimental conditions, such as button stiffness, the effector with which the participants execute the movement, and whether the response requires a button press or release (Coxon et al., 2007; Gronau et al., 2024; Weber et al., 2025). Notably, EMG partial responses do not appear on every successful stop trial. Electromyography-based indices of stopping speed only reveal when central drive is reduced at the level of the muscle, not the neural mechanisms underpinning this, nor the latency of stopping mechanisms upstream to muscle suppression (Raud et al., 2022). A benefit of this is that lapses of attention (the go stimulus failing to trigger the go response) are automatically excluded from calculations of stopping speed. A potential limitation is that trials in which the go process was suppressed before arriving at the muscle are also excluded, and it is ambiguous as to whether excluding nonmanifest stopping meaningfully biases this as an index of stopping speed. A recent study observed that both EMG-based measures—and SSRT—demonstrate low test–retest reliability, suggesting the speed of action cancellation (based on the measures we have to assess it) may lack trait-like stability (Thunberg et al., 2024). While this bears broad implications for studies that conceive stopping capacity as a trait-like quality, we also note that these measures showed high split-half reliability, supporting their usefulness when comparing between conditions within an experimental session. Notably, the reliability of EMG-based stopping indices under other conditions (e.g., when assessing stopping speed where baseline contraction is present in nonstopped effectors; De Havas et al., 2020, 2025) remains unexplored. In sum, despite the popularity of the SST, and the proliferation of more complex variants, there are still fundamental unanswered questions regarding how performance—even in simple SSTs—should be indexed and interpreted. Stop signal reaction time was initially conceptualised based on simple SSTs and assumes singular go and stop processes. This does not generalise well to complex stopping tasks (described below) where, for example, there are numerous go movements, or numerous types of infrequent stimuli (aside from the stop signal).

### The neural mechanisms of action cancellation

**Fig. 1:**
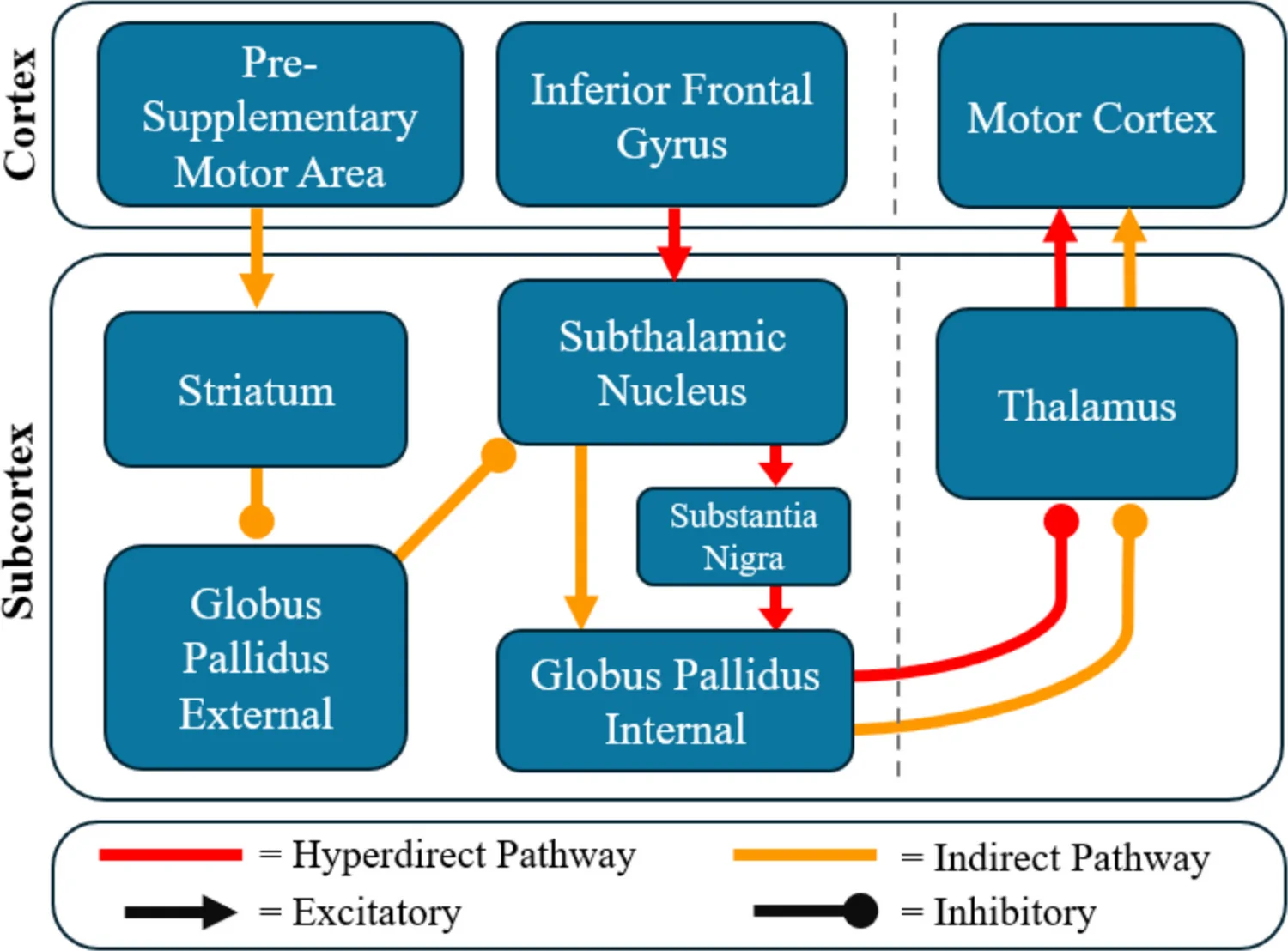
Corticobasal ganglia pathways thought to underpin action cancellation. *Note.* At rest, the basal ganglia (bottom left section of figure) exert a net inhibitory output, suppressing movement. The direct pathway (not pictured) culminates in a net *disinhibitory* effect, resulting in movement. The **indirect** pathway is thought to originate in the presupplementary motor cortex and, via a multisynaptic network, results in a net inhibitory effect on thalamic output. The **hyperdirect** pathway, like the indirect pathway, exerts a net inhibitory effect on thalamic output (Chen et al., 2020; Isherwood et al., 2025; Nambu et al., 2002) but involves a direct connection from the cortex to the subthalamic nucleus, allowing more rapid signalling to the motor cortex (for a detailed review of the basal ganglia circuitry, see Jahanshahi et al., 2015)

Two corticobasal ganglia pathways are thought to underpin action cancellation, although the functional contributions of each, and how these contribute to performance in stopping tasks, is the subject of ongoing debate. An overview of the key pathways is provided in Fig. 1. A current challenge in the literature is that the way these pathways should be reconciled with neurophysiological signatures of stopping is currently uncertain. One of the dominant models describing the function of these pathways—the “pause then cancel” model—posits that two mechanisms underpin action cancellation: a “pause” process (purportedly mediated by the hyperdirect pathway), which results in a transient, nonselective suppression of motor output associated with attentional capture, and a slower “cancel” process (mediated by the indirect pathway), which modifies or cancels the movement according to task demands (Diesburg & Wessel, 2021; Schmidt & Berke, 2017). By this account, these two processes can be teased apart and manifest differently in neurophysiological studies. A key challenge in the literature is trying to reconcile this account with observations at the level of the muscle, which predominantly suggest a single mechanism of stopping (Chaudhuri et al., 2025; Salomoni et al., 2025; although also see De Havas et al., 2025 for potential evidence of dissociable processes at the level of the muscle under specific conditions). Importantly, because inferences about the functions of neural mechanisms rely on the capacity of behavioural paradigms to isolate them, this debate is embroiled in questions regarding task design. Below, we begin by describing variations in simple stopping tasks, before moving on to more complex variants, where we discuss the current theoretical debates that motivate them, and make suggestions for future research.

## The stop signal task

### Simple vs Choice go responses in the stop signal task

**Fig. 2:**
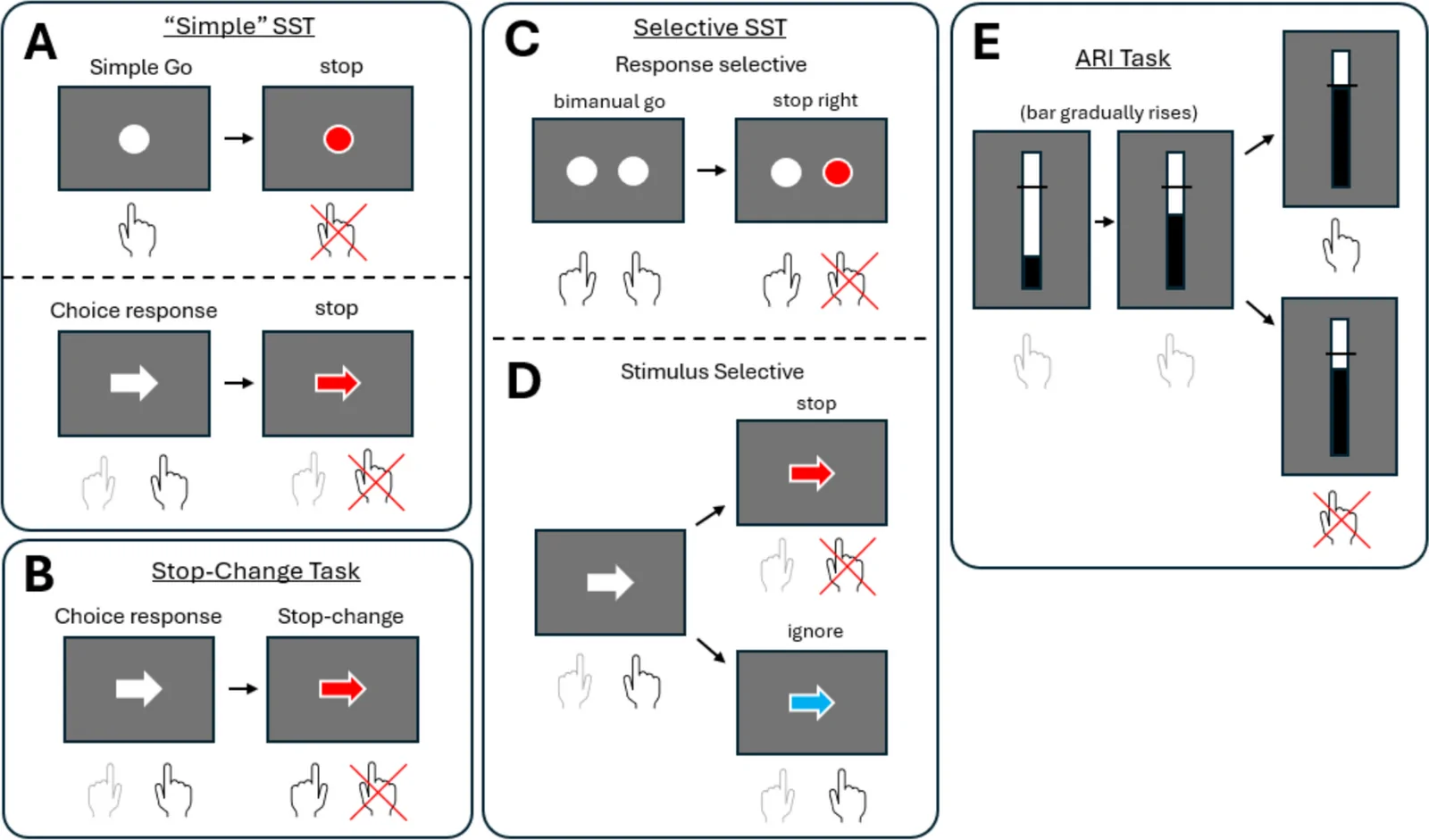
Commonly implemented stopping tasks. *Note.* Examples of the defining manipulations of each task. Implementation of these tasks in the literature (stimuli used, timings, effectors) is heterogenous. For all tasks, we depict an example of the required go response and what is required upon presentation of additional stimuli. **A)** Simple SSTs utilise a response either with, or without, a choice component. Stop signals indicate no response should be made. **B)** Stop-change tasks require the participant to inhibit their initial response and initiate another upon presentation of a stop-change signal. **C)** Response selective SSTs require that only one part of a multicomponent go response be inhibited. **D)** Stimulus selective SSTs require the participant to cancel responses to only one type of numerous possible infrequently occurring stimuli. **E)** ARI tasks feature a stimulus moving towards a target, indicating the required timing for the go response. The stop signal occurs prior to this (often conveyed by the moving stimulus stopping prior to reaching the target)

A graphical overview of key stopping tasks is presented in Fig. 2. Panel A represents examples of conventional, “simple” SSTs. These may require a button press with no choice component (a single button, with no selection between response alternatives required), or (more commonly) a choice response (with the go signal indicating which of two possible responses is required). Unsurprisingly, the go process is slower when a choice response is required, on account of participants needing to discriminate stimulus features and associate these with a specific behavioural response (Logan et al., 1984; Miller & Low, 2001). While early work suggested that SSRT is also slower in SSTs featuring a choice go response, compared with those requiring a simple response (Logan et al., 1984), to our knowledge there are no contemporary publications assessing this on a within-subjects basis either with behavioural or EMG-based measures of stopping speed.

Importantly, within-subject split-half internal reliability of SSRT estimates is greater for choice response SSTs than simple response SSTs (Leunissen et al., 2017), suggesting that, if selecting between the two, the former is preferable. This observation may relate to differences between these two task variants in the degree to which participants slow their go responses in anticipation of the stop signal: When expecting an upcoming stop signal, adjustments are made within inhibitory networks, to facilitate the potential upcoming action cancellation (Lavallee et al., 2014; Van de Laar et al., 2011). Excessive anticipatory slowing of the go response, and changes to the amount of slowing over time, can invalidate SSRT estimations (Verbruggen et al., 2013). Unlike tasks with a choice response, successfully stopping in SSTs that use a simple go response may *require* some degree of proactive slowing, on account of go responses being much faster without a decision-making component. In healthy young adults, electrophysiological measures of stopping speed typically a reveal suppression of muscle activity 160 ms following stop signal onset (Jana et al., 2020; Raud & Huster, 2017; Raud et al., 2022; Salomoni et al., 2025). Given mean simple reaction times in healthy young adults typically range from 200 to 230 ms (Woods et al., 2015), it is unsurprising that (to improve the chances of stopping successfully) participants adopt a response strategy with a greater degree of proactive slowing in SST tasks that lack a choice component. That is, even at very short SSDs, a fast go response will preclude successful stopping. As such, for traditional calculations of SSRT, choice responses are recommended (Verbruggen et al., 2019). Notably, responding to a choice reaction time task introduces additional cognitive processes: stimulus decoding, stimulus–response mapping, and response selection (Miller & Low, 2001). While this minimises the need for proactive slowing, it does introduce greater ambiguity regarding which of the previously noted processes is being interrupted (Aksiotis et al., 2023). Notably, this ambiguity does not undermine SSRT calculation provided independence between go and stop processes is assumed (i.e., it does not matter which stage the go process has reached, only whether it will be overtaken by the stop process; Logan et al., 1984).

### Stimulus modality in the stop signal task

Stop signal tasks most commonly involve visual go signals and either visual or auditory stop signals. This choice of modality for stop signals often seems quite arbitrarily decided, with different research groups appearing to preference a particular modality. Given differences in transduction and transmission times between visual and auditory information, it is unsurprising that sensory modality influences stopping speed (Bullier, 2001; Thorpe et al., 1996; Ulrich et al., 1998). Indeed, a large body of evidence suggests stopping is *faster* in response to auditory stimuli than visual stimuli (Morein-Zamir & Meiran, 2003; Ramautar et al., 2006; Van Der Schoot et al., 2005), although not all papers observe this (Ikarashi et al., 2022). Research using electroencephalography has suggested that this observation is primarily a result of bottom-up differences (i.e., faster auditory *c.f.* visual processing) rather than differences in top-down inhibitory processes (Carrillo-de-la-Peña et al., 2019), although top-down processes may also prime sensory specific cortices to facilitate rapid action cancellation (Kenemans, 2015).

Recent work has revealed that the faster stopping observed in response to auditory signals only occurs when a visual go signal is used (Weber et al., 2024b) and additionally that stopping to visual stop signals is faster (than stopping to auditory signals) when an auditory *go* stimulus is used. That is, stopping in cross-modal tasks is faster than stopping in unimodal tasks. This suggests some degree of psychological refractoriness in unimodal stopping tasks (i.e., greater processing delays as a result of shared sensory resources being used to process both go and stop; Wickens, 2008). It also indicates that, at least in part, there is a dependence between the go and stop process in unimodal tasks on account of competing sensory resources, creating further challenges for the horse race model underpinning SSRT, which assumes their independence (Kenemans, 2025). Taken together, results indicate that the sensory modality used in SSTs has a direct influence on stopping speed and this likely results from competing sensory processing resources influencing the salience of the stop signal.

In sum, the manner in which go response format (simple vs choice) and stop signal modality (auditory vs. visual) influence metrics of stopping performance remains incompletely understood. This creates challenges for generalisability and interpretation, even in simple stopping tasks. These methodological differences also permeate through complex tasks variants, which we describe below.

### “Complex” stop signal tasks

With the common variations of the simple SST described, we will move on to discuss more complex variants, explaining a) the key questions they address regarding the mechanisms of action cancellation and b) to what extent performance in these tasks is meaningfully relatable to those in simple stopping tasks.

### Stimulus selective stop signal tasks

In stimulus selective SSTs, one of two perceptually distinct types of infrequent signal may follow the go signal on any particular trial, although only one of these is associated with an imperative to stop (Fig. 2D; Bedard et al., 2002; De Jong et al., 1995; Van de Laar et al., 2011). The other (referred to variably as an “ignore,” “catch,” or “continue” signal), bears no behavioural imperative; the participant must try to ignore it and proceed with their response to the go stimulus. It has been argued that these tasks demonstrate greater ecological validity compared with the standard SST on the basis that most everyday scenarios that require stopping require differentiation between types of stimuli/events. For example, when hiking, we might need to rapidly differentiate between a snake on the track or an inanimate fallen branch that bears no risk. These tasks have been used as an alternative to standard SSTs to identify changes in stopping speed across the lifespan (Bedard et al., 2002; Van de Laar et al., 2011) and differences between healthy and clinical populations (Bedard et al., 2003). However, behavioural results in these tasks frequently demonstrate violations of the independent race model, invalidating traditional SSRT calculations. In particular, the requirement to discriminate between stop and ignore stimuli before stopping may interfere with (i.e., slow) the go process in stop trials (Bissett & Logan, 2014), especially when stimulus discrimination is difficult (Sánchez-Carmona et al., 2021). We also note that the traditional horse-race model was designed to capture go and stop trials only. Trials involving the presentation of an additional stimulus that does not require stopping are not accommodated within this framework. Given that violations of the horse-race model can result in substantial misestimation of SSRT (Verbruggen et al., 2013, 2019), SSRT estimation is not recommended under such conditions or should at least be interpreted with caution.

Stimulus selective SSTs have also been implemented in attempts to differentiate between involuntary inhibition associated with attentional capture and voluntary action cancellation (Sharp et al., 2010). A growing body of research demonstrates that a generalised global inhibitory response occurs upon attentional capture to unexpected events (Wessel & Aron, 2017). Brain imaging research has implicated many of the same regions thought to be involved in reactive stopping, such as the right IFG and subthalamic nucleus (Fig. 1; Sebastian et al., 2021). This has contributed to the pause-then-cancel model of action cancellation (introduced above), which decomposes action cancellation into two distinct components: a nonselective suppression of motor output associated with attentional capture (pause), and a slower, voluntary removal of drive from basal ganglia output (cancel; Diesburg & Wessel, 2021; Schmidt & Berke, 2017). By this account, because SSTs feature an infrequent “unexpected event” (i.e., the appearance of the stop signal), they may conflate the pause and cancel process, both of which result in suppression of motor output. By potentially triggering the pause process, but not the cancel process, ignore trials have been used in attempts to tease these apart. Indeed, ignore stimuli have been shown to elicit response slowing and suppression of muscle activity (Ko & Miller, 2013; Wadsley et al., 2023; Weber et al., 2023). Furthermore, brain stimulation research demonstrates neural suppression of the corticospinal tract occurring similarly for stop and ignore signals up until ~ 200 ms following the presentation of the stop/ignore signal, at which point suppression is released in ignore trials, but not stop trials (Tatz et al., 2021), supporting the idea of a generic mechanism responding to any unexpected stimulus. As further support for this interpretation, brain imaging contrasts between stop and ignore trials have demonstrated activity in the pre-SMA to be specific to stop trials, while IFG activity is common to both stops and ignores (Kemp et al., 2024; Sharp et al., 2010), consistent with the pause-then-cancel framework, which posits that the pause process originates in the IFG and the cancel process in the pre-SMA (Diesburg & Wessel, 2021).

Nonetheless, while some papers have conceptualised ignore trials as isolating the pause process (Wadsley et al., 2023), the inclusion of a second type of infrequent stimulus also introduces an additional task requirement: rapid stimulus discrimination during a task where the participant knows there is the potential need to stop. In this context, participants may adopt a strategy of stopping to all infrequent stimuli, before deciding to initiate a response to ignore trials (Bissett & Logan, 2014). Given this, stimulus selective SSTs cannot be said to *specifically* isolate attentional capture to infrequent stimuli. Indeed, when ignore trials are included outside a stopping context (in a task where other infrequent stimuli cue the enaction of a second response, rather than cancellation of a response) the number of partial responses that can be recorded with EMG, and associated behavioural delays, are reduced (Weber et al., 2023). As such, despite their increasing use, it is currently unclear what process are being initiated by ignore trials in stimulus selective SSTs, nor how contextually specific the observations from these tasks are. Future research using brain imaging and brain stimulation techniques directly comparing equivalently salient and frequent ignore signals both within and outside of a stopping task would help to tease apart what additional processes are recruited to ignore trials in stimulus selective SSTs.

In sum, stimulus selective SSTs have contributed to the recognition that many of the purported signatures of stopping manifest in contexts beyond the voluntary cancellation to a stop signal. However, fundamental aspects of the task design preclude valid SSRT estimates (via traditional methods), and caution should be applied regarding the interpretation of these tasks as a means of isolating attentional capture.

### Response selective stop signal tasks

Response-selective SSTs (also referred to as motor-selective SSTs; Fig. 2C) feature stop trials that require the participant to inhibit only part of a multicomponent go response, while continuing with another component (e.g., stopping only one hand during a bimanual movement; Coxon et al., 2007). Given real-world instances of reactive action cancellation typically involve continuation of some movement rather than complete movement cessation (e.g., continuing to turn the steering wheel while cancelling a foot movement to press a pedal), response-selective stopping tasks are (much like stimulus selective SSTs) often argued to have greater ecological validity than standard SSTs. They also allow for insights into whether the neural mechanisms mediating action cancellation have been applied specifically to the effector that was cued to stop or whether they have influenced motor output more broadly. For instance, brain stimulation studies provide evidence that action cancellation has broad effects on motor output, coinciding with suppression of motor output in task-irrelevant muscles on the same hand (Van Den Wildenberg et al., 2010), homologous muscles on the nonresponding hand (Coxon et al., 2006), and even leg muscles, despite these not being used in the task (Greenhouse et al., 2012). This global suppression is also observable via EMG, as a transient suppression in muscles that are maintaining ongoing tonic activity (De Havas et al., 2025; Mills et al., 2025; Weber et al., 2025), or as a transient reduction in force, if ongoing force is applied to force sensors (Rangel et al., 2024).

Notably, in stimulus selective SSTs, the response time of the continuing hand (the hand that was *not* required to stop) is typically slower^4^ than the RTs that occur in the absence of a stop signal (Cai et al., 2011; Claffey et al., 2010; Coxon et al., 2007; Drummond et al., 2018; Raud & Huster, 2017). This delay has been termed the “stopping interference” effect (Aron & Verbruggen, 2008; Coxon et al., 2007), although is also referred to as “stopping delay” (Gronau et al., 2024). The precise neural mediators of this behavioural phenomenon are a topic of ongoing debate (Wadsley et al., 2022).

Early research using response-selective SSTs manipulated the predictability of the side that would be required to stop, allowing for insight into whether the delays arose due to uncertainty regarding which side a movement would be required on (Aron & Verbruggen, 2008). Unsurprisingly, when the participant has advance knowledge regarding the side of the stop signal, the stopping interference effect is reduced (Aron & Verbruggen, 2008). Notably, this was observed alongside an *increase* in SSRT (Aron & Verbruggen, 2008; Claffey et al., 2010). In combination, these findings contributed to one of the dominant models of selective stopping, which posits that global stopping is mediated by the rapid hyperdirect pathway while response-selective stopping is mediated by the slower indirect pathway (Aron, 2011; Fig. 1). More recently, this account of selective stopping has come into question. For example, recent research using cognitive modelling and EMG-based measures (which preclude the need to assume that traditional SSRT calculations generalise to a response-selective stopping context) revealed that, while advance knowledge of the specific stopping demands reduced the stopping delay, it did not influence the speed of the stop process (Salomoni et al., 2023). Further research also observed that stopping speed (determined by EMG measures and cognitive modelling) was equivalent for global and selective stopping, establishing a unified model for global and selective stopping, referred to as the Simultaneously Inhibit and Start (SIS) model (Gronau et al., 2024). By this account, the same stopping mechanism is used for global and selective stops (or at least, if distinct mechanisms are used, these influence motor output with the same latency).

An account of stopping delays based on the pause-then-cancel model (introduced above) would suggest they result from the globally applied pause process (mediated by the hyperdirect pathway; Fig. 1) transiently inhibiting all motor output before selective cancellation is applied on the side that needs to be cancelled (Wadsley et al., 2023). However, this interpretation means that the pause process coincides with the successful cancellation of a movement, and the functional distinction between this and the cancel process becomes unclear. That is, if the pause process is sufficient for successful action cancellation, the cancel process seems redundant (Chaudhuri et al., 2025). More recently, proponents of the pause then cancel account have suggested a “pause-then-retune” variant, swapping out the cancel for a more generic “retune” that relies on the same neural underpinnings but facilitates either rapid action cancellation or action reprogramming (Hervault et al., 2025; Tatz et al., 2021). This could allow for an account of response-selective stopping whereby the ongoing movement is represented as a result of the “retune” process, while the cancellation of movement represents the pause process. This may be partially reconcilable with the SIS model, given that the same mechanism (labelled a “pause” by the pause-then-cancel/retune account) may underpin stopping in both global and selective stops (Gronau et al., 2024). Notably, the SIS model describes the response that occurs in the ongoing hand as a *distinct* go process—triggered by the stop signal. Whether this can be accurately described as the result of a “retune” is a question regarding whether the neural underpinnings of this movement rely on specialised networks (the indirect pathway) or the same neural processes that underpin the initial voluntary go response. We note that the speed of reprogramming in response selective SSTs is (unlike the speed of stopping) modifiable based on prior information and task goals (Salomoni et al., 2023), and that while this plausibly can be called a “retune” process, little is known about the neural mechanisms that mediate this proactive adjustment. For instance, facilitation of action reprogramming/retuning is achieved, at least in part, by preparatory modulation of the corticospinal tract (Claffey et al., 2010; Kemp et al., 2025), something that the pause-then-retune account does not currently encapsulate. We expand more on how the pause then cancel/retune account may be reconciled (or not) with behavioural and physiological findings in the concluding section.

With regards to methodological inconsistencies in response-selective SSTs, we note that contrasting observations in response-selective stopping using traditional SSRT calculations may also be due to the nature of the go response. In contrast to most simple SSTs, response-selective stopping tasks typically do not require a choice response to the go stimulus. That is, a bimanual response is required on all go trials. Given greater degrees of proactive slowing are demonstrated when the go response does not require a choice (see above section on simple stopping tasks), and this can invalidate SSRT calculations (Verbruggen et al., 2019), traditional calculations of SSRT are particularly problematic in these tasks (Leunissen et al., 2017). While EMG-based measures provide an alternative, they are not immune to this problem: for the offset of muscle activity to be determined, a go process needs to have been initiated in the first place. As such, if responses are being initiated later (due to strategic slowing), this will bias stopping measures (Raud et al., 2020). Notably research comparing global and selective stops using EMG-based measures has, to our knowledge, only used simple, rather than choice, go responses (Gronau et al., 2024; Raud et al., 2022). As such future research implementing contemporary measures of stopping (cognitive modelling and EMG), comparing response-selective and global stops with and without choice responses would improve the generalisability of these claims.

In sum, while response-selective stopping tasks have allowed for insights into the selectivity of the stop mechanisms, fundamental questions remain regarding a) the validity of the indices of stopping in these tasks; b) the precise impacts that deviation from the design in simple SSTs has on the generalisability of results; and c) the neurobiological underpinnings of performance in these tasks. Ultimately, the roles of the pause and cancel processes in response selective stopping are not easily reconcilable with models based on EMG and behavioural data (the SIS model described above). This represents a current key area for future research in selective stopping.

### Stop-Change tasks

The stop-change task (Fig. 2B) is an extension of the regular SST in which, rather than simply cancelling a response when the stop signal is presented, participants must replace it with another (distinct) movement (Logan, 1994). Like the other complex stopping tasks described above, it has been argued that this task has greater ecological validity, as in real-world situations we rarely abandon obsolete actions without immediately initiating another (Boecker et al., 2013). This task differs from response-selective stopping tasks as the go does not require a multicomponent response, although is similar in that a response is still made following the inhibition of an initial movement.

Traditional methods of SSRT calculation have been used in stop-change tasks, based on the notion that the stop and change responses are performed in serial, and that the additional movement after the stop does not interfere with the underpinning race model (Verbruggen et al., 2008). Via this method, these tasks have been used to identify inhibitory deficits in clinical populations, especially children and adults with ADHD (Bekker et al., 2005; Boecker et al., 2013; Geurts et al., 2004), as the requirement to make a movement after action cancellation may prevent lapses of attention (failure to initiate a response) being classified as successful stopping. Interestingly, when implemented in study designs as distinct tasks, SSRT in stop-change tasks has been reported as slower than that in simple SSTs (De Jong et al., 1995; McClure et al., 2005), although when these tasks are integrated, such that stops and changes are required on different trials within one experimental block, this difference does not persist (Drueke et al., 2010). This has led some authors to speculate that slower stopping in stop-change tasks is mediated by task difficulty and working memory demands, rather than differences in stopping mechanisms due to the subsequent requirement to change (Boecker et al., 2013). Knowing that a subset of the infrequent signals will require an updated response also introduces a rapid stimulus discrimination component to the task and may influence response preparation. fMRI research contrasting stop and change trials revealed only significant differences in regions that could be explained as resulting from the additional requirements of enacting a second (changed) response,^5^ supporting the notion that the same stopping mechanism is implemented in each task (Boecker et al., 2011). Notably, this research used a mixed design, with stop and change trials randomly occurring within the same experimental blocks, and the influence of rapid stimulus discrimination, and whether stopping mechanisms should be considered the same in SSTs and stop-change tasks, remains ambiguous. Future research using brain imaging techniques on a within-subjects basis, comparing block and trial-level implementations of the stop and stop-change tasks, would help to resolve some of the ambiguity in the underlying processes involved.

More recently, stop-change tasks have been used to provide putative evidence for an updated version of the pause-then-cancel model of stopping; the “pause-then-retune” account (introduced above). By this account, following a generic hyperdirect-pathway-mediated pause, the indirect pathway facilitates either the removal of drive from corticobasal-ganglia circuits (a cancel), *or* the rapid adjustment of motor output, to modulate motor plans (a “retune”: Hervault & Wessel, 2025; Tatz et al., 2021). The authors posit that particular electroencephalography signatures (specifically the frontocentral P3) reflect the cancel/retune process and that these seem to “scale” depending on the degree of retuning required trials (Hervault & Wessel, 2025). Unexpectedly, and in contrast to past research, the authors also observed *faster* stopping (as indexed by traditional SSRT calculations and EMG-based measures) in stop-change trials compared with stop trials (Hervault & Wessel, 2025). This observation was possibly confounded by stimulus salience effects. Specifically, stop-change trials in this task featured an additional visual stimulus in a different location to the go stimulus (in contrast to stop trials, which just featured the go stimulus changing colour). Indeed, presenting a stop signal in a different location to the go signal enhances stopping speed (Friehs et al., 2024), which may account for the faster stopping in stop-change trials. Furthermore, increasing the salience of the stop signal can influence the frontocentral P3 ERP amplitude (Kenemans et al., 2023). As such, the question of whether the pause-then-retune model is supported has become entangled with questions around stimulus salience effects, and the correct interpretation of these findings is therefore ambiguous. This highlights a broader challenge in the stopping literature, which is that the requirement to communicate complex imperatives to the participant requires increasingly complex stimuli, but our understanding of stimulus effects (not just on behaviour but other neurobiological signatures) is incomplete, or is unaccounted for. Accordingly, as with the other varieties of complex stopping task described above, we believe that the literature would benefit from clear within-subject comparisons between simple SSTs and stop-change tasks that carefully control for stimulus effects.

### Anticipated response inhibition tasks

Like the SST, the anticipated response inhibition (ARI) task is composed of both go trials and stop trials. However, in a go trial, the participant must enact a response at a *predictable* time, when a moving indicator intersects a stationary target (Coxon et al., 2006; He et al., 2022; Slater-Hammel, 1960). Commonly, this moving indicator is a vertical bar “filling” (i.e., changing colour) from the base upwards, at a constant rate.^6^ The time point at which this rising colour change intersects a horizontal target line informs the participant when their response should be registered (Fig. 2E). Accordingly, participants are able to prepare their response, and precisely time its occurrence to coincide with the alignment of stimuli. In these tasks, the stop signal occurs *prior* to the point where the moving indicator intersects the target, such that the participant must attempt to cancel their prepared response. The stop signal is commonly represented by the filling bar halting its upward progress before it reaches the target line.

ARI tasks have been used to assess changes to inhibitory networks in clinical populations (Van Rooij et al., 2015; Zandbelt et al., 2011) and during development (Van Hulst et al., 2018; Vink et al., 2014). While SSRT may be calculated in ARI tasks (He et al., 2022), variants of this task demonstrate violations of the assumptions of independence between the go and stop process (Wadsley et al., 2022; Weber et al., 2024a). Additionally, the horse-race model assumes that the go stimulus is consistently present throughout the trial, while many versions of the ARI represent the stop signal as being a *cessation* of the go stimulus, again causing problems with the estimation of SSRT (Matzke et al., 2018). Furthermore, having the stop signal occur prior to the go has been shown to alter participants’ perceptions of the passage of time in trials with a stop signal (on account of the “filled-interval” illusionThomas & Brown, 1974; Wearden et al., 2007), influencing the go process in stop trials and invalidating traditional SSRT, although novel modelling approaches have provided solutions to these concerns (Matzke et al., 2021).

A potential benefit of the ARI task is that instructing the participant to time their response to coincide with a predefined timepoint minimises strategic slowing of the go response in anticipation of the stop signal (Verbruggen et al., 2013). This removes the positive skew that is typical of speeded reaction times. This reduction of variability of the go response has made these tasks particularly popular in brain stimulation research seeking to probe modulation of motor networks prior to response initiation. For example, highlighting the role of M1 intracortical inhibition in action cancellation (He et al., 2019),^7^ and the role of interhemispheric communication during simple and response-selective stopping (MacDonald et al., 2021). We also note that these mechanisms, while demonstrably implicated in inhibition, are typically not included in current attempts to provide a unified account of action cancellation (Diesburg et al., 2021), typically seeming to exist in parallel.

Behavioural research using conventional SSRT calculation in ARI tasks and simple SSTs has observed faster stopping in ARI tasks, relative to simple SST variants (Leunissen et al., 2017). However, more recent research, using EMG based measures to index stopping speed, has revealed no difference between stopping in an SST and ARI task (Weber et al., 2024a), although this paradigm implemented ARI tasks and SSTs that required both response and stimulus selective stopping. Accordingly, as with many comparisons across the inhibition literature, it is currently unclear here whether differences arise due to the *indices* used to assess stopping, or as a result of task context/conditions.

### Conclusions and recommendations for future research

There is currently a lack of consensus regarding how action cancellation should be elicited and how it should be indexed (Thunberg et al., 2024). While some attempts have been made to establish overarching conventions for simple SSTs (Verbruggen et al., 2019), these tasks variably employ auditory or visual stimuli, choice or simple responses, or other methodological differences, which meaningfully influence performance. Furthermore, the precise relationship between indices of stopping (whether behavioural or physiological) and brain activity, remains ambiguous (Chaudhuri et al., 2025; Diesburg & Wessel, 2021; Huster et al., 2022). Complex stopping tasks have the potential to advance theoretical and anatomical models of action cancellation as well as increase ecological validity. However, they commonly introduce additional stimulus effects and increased cognitive demands beyond those which they seek to target (e.g., increased working memory demands as a result of additional response-stimulus mappings; Aziz-Safaie et al., 2024). Critically, much of the past research assessing performance in these tasks uses traditional SSRT measures, which are based on a conception of action cancellation developed for the simple SST. This has led to reports of differences in stopping speed between, for example, ARIs and SSTs (Leunissen et al., 2017), global and response selective stopping (Aron & Verbruggen, 2008), and stopping with and without informative cues (Claffey et al., 2010). Above, we described how these differences have not manifested when EMG based measures and/or cognitive modelling techniques have been implemented (Gronau et al., 2024; Weber et al., 2024a, 2024b; Salomoni et al., 2023). Findings derived from these techniques point towards a generic reactive stopping mechanism that generalises across many task contexts (Chaudhuri et al., 2025; Salomoni et al., 2025). Interestingly, this is not easily reconcilable with popular two-stage models of action cancellation. While there is robust evidence that a pause response exists in response to attentional capture (Wessel & Aron, 2017), the role this plays in stopping paradigms is not clear. It seems likely that either this same mechanism can be up-regulated to facilitate stopping in contexts that require it and/or a distinct automatic fast-acting mechanism operates in contexts where stopping is required. Reconciling the observations from modelling and physiological approaches with underpinning neurobiological mechanisms represents a current key challenge in the field. Multimodal research testing possible associations between muscle level observations (partial responses) and brain imaging or electrophysiological results (contrasting successful stops with and without partial responses) could help to reconcile stopping at the muscle with the upstream neural mechanisms. Further research is also required to provide support for (or challenge) the functional distinctions that two-stage models propose and to understand how they might be reconciled with physiological signatures of stopping. Comparing how these manifest to equivalent stimuli in and outside of stopping tasks represents a key path forward. For instance, to what degree is corticospinal excitability suppressed following infrequent but salient ignore signals outside of a stopping context and is this comparable to ignore signals *within* a stopping context (as seen in Tatz et al., 2021)? We also note that advancement of this debate may draw on tasks other than SSTs. The suppression of movement often occurs, not in response to discrete events in our environment but as a result of internal decision-making processes (Stone et al., 2022). Recent work highlights the potential to investigate self-generated cancellation of movement using force sensors (Foerster et al., 2022), mouse tracking (Tonn et al., 2025), and EMG (Weber et al., 2026). Given that these changes involve the cancellation of movement, without attentional orienting to an unexpected stimulus, they may provide another means of dissociating the inhibitory signatures associated with each. Coupling these techniques with brain imaging (e.g., fNIRS) or brain stimulation techniques would provide further insights here.

Conventional stop signal tasks are in themselves highly artificial, placing the participant in a context that is far removed from real-world stopping situations. While introducing additional perceptual components and more complex response mappings can ambiguate interpretation, they can also improve ecological validity, facilitating the translation of response inhibition to real-world contexts (Hannah & Aron, 2021). While we have focused on task variants that we believe are currently driving theoretical accounts of stopping, SSTs have been used in combination with perceptual decision tasks (Jahfari et al., 2015), reward/punishment paradigms (Verbruggen & McLaren, 2018), colour Stroop tasks (Kalanthroff et al., 2013), and Erikson Flanker tasks (Bundt et al., 2025; Healey et al., 2024). Stop signal tasks have also found their way into the gamification literature, with findings that gamification may increase motivation, but result in slower stopping latencies compared with traditional simple SSTs (Markiewicz et al., 2024). It is our hope this review provides a cautionary tale regarding the interpretation of results, and validity of traditional methods of SSRT calculation (i.e., the integration method and mean method; Verbruggen et al., 2019) in complex stopping contexts, where the traditional horse-race model does not apply. Where possible, we recommend the use of EMG-based indices in complex stopping tasks, rather than SSRT calculations.

As a concluding note, we emphasise that, given that the above complex stopping tasks all provide opportunities to address current theoretical questions in the literature, there is currently high need for research allowing for within-subject comparisons across tasks. It is often unclear, when comparing results from different stopping tasks, what should be interpreted as stimulus effects, cohort effects, or a true difference in how the nervous system implements action cancellation. Without establishing a clearer understanding of the way stopping indices (both behavioural and physiological) differ across complex stopping tasks, the literature risks accumulating large bodies of task-specific research that fail to inform our understanding of action cancellation outside the laboratory.

## Data Availability

Not applicable.

## Footnotes

1 Notably, some research has employed hybrids of the two tasks (i.e., a majority of go trials, a portion of stop trials—in which the stop signal occurred after a delay, and a portion of no-go trials—in which the stop signal occurs synchronously with the go, Schachar et al., 2010; Swick & Ashley, 2020).

2 The “integration method” is commonly used to calculate SSRT (Verbruggen et al., 2019). This method involves using the distribution of response times on go trials—assuming these to be the same as the distribution of go process finishing times on stop trials. A point along this distribution, representative of the threshold at which stopping can be achieved, is identified (e.g., if someone failed to stop on 40% of stop trials, and there were 100 go trials this would be the 40th fastest RT). Mean SSD is then subtracted from this value (as SSD is when the stop signal appears and the stop runner starts) to give SSRT.

3 Notably, this is not the only interpretation of partial responses. In contexts where participants must make a decision between competing response options, or where stimuli introduce conflict between choice options (such as the Erikson flanker task), partial responses have previously been interpreted as a “change of mind”—occasions where a response was selected and initiated at the level of the muscle, but abandoned prior to a behavioural response being generated (a button press), before another response was made instead (Servant et al., 2014; Stone et al., 2022; Weber et al., 2026).

4 Usually by ~ 100 ms, although this is influenced by individual differences (Majid et al., 2012), and under very specific conditions and task designs, may be eliminable (Xu et al., 2015). Nonetheless, it has been argued that the degree of contextual specificity required to prevent the occurrence of stopping delays (and the otherwise robust findings of this effect) suggest that it is not just an artifact of the SST but rather a reflection of inherent central nervous system limitations.

5 In particular, contrasts between stop and change trials revealed greater activity in change trials in the motor cortex, premotor cortex, postcentral gyrus, and inferior parietal lobe. Notably, not in regions typically associated with action cancellation (right IFG, pre-SMA; Fig. 1).

6 However, some earlier research used a clock-like stimulus with a rotating pointer (Carlsen et al., (2008; Coxon et al., 2006).

7 The degree of suppression to corticospinal activity induced by GABAergic interneurons in M1 can be probed by administering two TMS pulses in rapid succession: a priming pulse to trigger these interneurons, and a test pulse—for which muscle activation is measured with EMG. The amplitude of the test pulse can be compared to muscle activation without a priming pulse (Sohn et al., 2002). Larger reductions in amplitude following a priming pulse are associated with improved stopping ability.
